# Nanomedicines: A Potential Treatment for Blood Disorder Diseases

**DOI:** 10.3389/fbioe.2019.00369

**Published:** 2019-11-28

**Authors:** Nan Zhang, Ming-Yuan Wei, Qiang Ma

**Affiliations:** ^1^Chinese Academy of Inspection and Quarantine, Beijing, China; ^2^School of Life Science and Medicine, Dalian University of Technology, Panjin, China; ^3^Texas Commission on Environmental Quality, Austin, TX, United States

**Keywords:** nanomedicine, blood disorder diseases, artificial blood components, treatment, emergency blood supply

## Abstract

Blood disorder diseases (BDDs), also known as hematologic, is one of the diseases owing to hematopoietic system disorder. Chemotherapy, bone marrow transplantation, and stem cells therapy have been used to treat BDDs. However, the cure rates are still low due to the availability of the right type of bone marrow and the likelihood of recurrence and infection. With the rapid development of nanotechnology in the field of biomedicine, artificial blood or blood substitute has shown promising features for the emergency treatment of BDDs. Herein, we surveyed recent advances in the development of artificial blood components: gas carrier components (erythrocyte substitutes), immune response components (white blood cell substitutes), and hemostasis-responsive components (platelet substitutes). Platelet-inspired nanomedicines for cancer treatment were also discussed. The challenges and prospects of these treatment options in future nanomedicine development are discussed.

## Introduction

Blood disorder diseases (BDDs), also known as hematologic, is one of the diseases owing to hematopoietic system disorder. BDDs can be broadly classified into three categories, red blood cell disease, white blood cell disease, and platelet disease. Among BDDs, iron deficiency anemia, leukemia, hemophilia, and malignant lymphoma are most widely known. Taking leukemia as an example, the early stage is extremely difficult to cure, resulting in high mortality rate. In 1948, Farber et al. realized a temporary remissions in acute leukemia, providing a promising direction for leukemia treatment (Farber and Diamond, 1948).

Researchers discovered that chemotherapy may be an effective means of treating BDDs with the high risk of causality due to immune system damage. The similar challenge exists in the bone marrow transplant treatment, which transplants normal human hematopoietic stem cells into the patient and rebuilds the patient's immune and hematopoietic system. A matching donor is rare to find, and the treatment cost is not affordable. Hematopoietic stem cells are rich in cord blood (CB), which could be considered as a bone marrow substitute. CB cells can be used for infusion therapy in special situations, because it allows a certain degree of leukocyte antigen mismatch. Patient survival rate could be improved by umbilical cord blood transplantation, such as the injection of two units of cord blood cells, or *ex vitro* expansion of cord blood cells (Pelosi et al., 2012). To date, this technology has been used for BDDs treatment, but some patients died of bacterial infection due to the insufficient doses (Maeda et al., 2005).

A great variety of methods for BDDs treatment have been developed in recent years. For example, as for severe thalassemia treatment, deferoxamine, or deferiprone is often used as a chelating agent to bind unstable iron to eliminate a series of effects from excessive iron content (Maggio, 2007). It was found that rituximab, an anti-CD20 monoclonal antibody, specifically depletes B cells, and reduces their damage to blood proteins or cells (Barcellini and Zanella, 2011). In addition, the modification or deletion of the telomere gene was found beneficial for the treatment of hematological diseases resulting from random or malignant transformation of hematopoietic function due to telomere shortening (Allegra et al., 2017). Genetic engineering has been attracted increasing attention for BDDs treatment. Townes et al. derived embryonic stem cells and then performed homologous recombination *in vitro* to change the pathogenic gene of sickle-shaped anemia, replacing the normal gene copy βββA with the disease-causing gene βββS (Townes, 2008). The diseased mice returned to normal and produced high levels of human hemoglobin. Osborn et al. efficiently induced double-stranded DNA breaks by the clustered regularly interspaced short palindromic repeats/associated protein 9 (CRISPR/Cas9) method, specifically editing clinically relevant T cell receptor alpha constant (TRAC) gene targets that are highly correlated with leukemia (Osborn et al., 2016). Compared with patients with non-hematologic malignancies, hematological malignancies, such as chronic lymphocytic leukemia, acute lymphoblastic leukemia, and non-Hodgkin's lymphoma, chimeric antigen receptors T cells (CAR T), had shown higher overall response rate and complete response rate.

Nanomedicine is a new branch of medicine that applies nanotechnology to traditional medical development. Nanomedicine focuses on designing a specific combination of nanomaterial (e.g., nanoparticle, nanocarrier, and nano-vesicle) and small molecules to conceive a biocompatible carrier for delivering drugs to cancer sites effectively (Wicki et al., 2015). The effective component could be a polypeptide, a protein, a nucleotide, or a small molecule drug. Compared with traditional medicines, nanomedicine appears to be able to avoid the body's defense mechanisms, reduce the clearance rate, prevent tissue damage through regulated drug release, and improve the pharmacokinetics and biodistribution of the drug. The nanoscale component to build a nanomedicine include: polymer nanoparticles (NPs), liposomes, metal NPs, carbon nanotubes, and molecularly targeted NPs. For instance, polymer nanoparticles are often composed of hydrophobic core and hydrophilic shell, on which specific targeting moieties were coated. Quintana et al. attached folic acid, methotrexate and fluorescein to polyamidoamine dendrimer, and the targeted delivery improved the cytotoxic response of the cells to methotrexate 100-fold over free drug (Quintana et al., 2002). In addition to conventional intravenous and oral medications, lung infection disease was treated by inhalation of NPs as an adjuvant therapy (Blum et al., 2014; Jurek et al., 2014). Marrache et al. used a targeted high-density lipoprotein (HDL)-mimicking NP with contrast agents to detect vulnerable plaque and initiate preventative therapy for atherosclerosis (Marrache and Dhar, 2013).

By 2016, 14 nano-drugs for clinical treatment was approved by U.S. Food and Drug Administration (FDA). In addition, FDA approves 18 nanomedicines for cancer chemotherapy in clinical trials, 19 nanopharmaceutical formulations for being developed and clinically tested, and 15 antibacterial nano-formulations for being developed (Caster et al., 2017). The development of common clinical nano-formulations appear to reduce toxicity rather than improve efficacy (Caster et al., 2017). The cytotoxicity and the stability in complex biological environments are still under debate and limit the progresses of nano-drug development (Lim et al., 2016). On December 21, 2018, FDA approved the first treatment for rare BDDs by granting Elzonris (tagraxofusp-erzs) infusion for the treatment of blastic plasmacytoid dendritic cell neoplasm (BPDCN) in adults and in pediatric patients (2 years or above) (FDA, 2018). Tagraxofusp-erzs is a cytotoxin that a protein is comprised of human IL-3 and truncated diphtheria toxin (DT), and it targets cells that express CD123, the alpha chain of the IL-3 receptor, which is overexpressed in BPDCN (Jen et al., 2019). The drug is preferentially accepted by CD123-overexpressed cells and initiates irreversible protein synthesis to cause cell death.

Despite of the excitement of the emerging of above-mentioned nanomedicines, direct blood transfusion of whole blood is preferred, if available, to efficiently treat BDDs. However, the storage of whole blood is challenging, due to short shelf life and the risk of infection by pathogenic bacteria (Hess, 2006; Greening et al., 2010). Most of the whole blood in the blood bank are donated by volunteers. At the beginning of the twentieth century, private blood sales led to the ravage of Human Immunodeficiency Virus (HIV) and Hepatitis B Virus (HBV) as a result of the lack of general health knowledge and safety awareness, especially in some economically backward developing countries. Taking China as an example, with the development of the country's economy, the need of blood donation, according to the National Health Service of China, has become desperately larger, but the public awareness of blood donation still falls behind. The blood banks' inventory became in shortage. Whole blood transfusion is often used for the patients after major surgeries or with BDDs, such as acute anemia. Therefore, the synthesis of blood substitutes as a supplement to the natural blood bank has become an urgent need.

Herein, we demonstrate recent advances in artificial blood components: gas carrier components (erythrocyte substitutes), immune response components (white blood cell substitutes), and hemostasis-responsive components (platelet substitutes). Moreover, the development of platelet-inspired nanomedicines was discussed. The challenges and prospects of these treatment options in future development are discussed.

## Nanomedicines for the Gas-Carrier Component

### Hemoglobin-Based Red Blood Cells Substitutes

The three key components of human blood are red blood cells (RBCs), white blood cells (WBCs), and platelets. The main function of RBCs is to serve as a gas carrier component for transporting oxygen and carbon dioxide. As discussed, RBC substitutes may be an alternative solution for emergency blood transfusion for BDDs treatment. The advantages of RBC substitutes include: (a) no surface antigens; (b) more convenient to store than natural blood; and (c) function as effectively as hemoglobin.

More than three decades ago, researchers extracted RBCs from expiring human or animal blood samples, broke the cells and centrifuged, and collected cell-free hemoglobin (Kothe et al., 1985), which was considered as an early version of RBC substitute. This hemoglobin solution showed the capacity of expanding oxygen delivery (Kaplan and Murthy, 1975), whereas the short effective time led to hypoxia-induced poisoning of liver, kidney, brain, and other organs (Friedman et al., 1978, 1979; White et al., 1986).

Chemical crosslinking of hemoglobin monomers was reported to eliminate the adverse effects of hemoglobin. For example, Biro et al. tested the effects of whole blood, unmodified stroma-free hemoglobin solution (SFHS), and partially cross-linked hemoglobin solution on coronary vessels in anesthetized open-chest dogs (Biro et al., 1988). The results showed that unmodified SFHS significantly caused vasoconstriction compared with whole blood perfusion. Blood vessels do not shrink when SFHS is subjected to pyridyloxylation (partial crosslinking). Infusion of adenosine in the coronary artery did not improve this condition. The results indicate that unmodified hemoglobin preparations cause coronary artery contraction in dogs and have an effect on their normal blood circulation (Biro et al., 1988). When the RBC lysate is covalently linked by adenosine triphosphate (ATP) or pyridoxal phosphate and purified by agarose nucleophilic chromatography, it can cause vasoconstriction of the coronary artery. This may be caused by the covalent modification, or that nucleophilic chromatography removes membrane phospholipids and denatured protein aggregates (Vogel et al., 1987; Lang et al., 1990).

Diaspirin cross-linked hemoglobin (DCLHb) is a modified hemoglobin that the two α helix units were crosslinked while the molecule maintains a tetramer conformation (the structure is shown in Table 1). DCLHb had shown a strong oxygen carrying capacity. DCLHb was in the treatment of severe traumatic hemorrhagic shock: a randomized controlled efficacy trial. Early experiments showed that DCLHb was well-tolerated and had shown no obvious functional disorders or toxicity (Przybelski et al., 1999). However, Sloan et al. found that the input of DCLHb resulted in an increase in patient mortality compared to the control group (Sloan et al., 1999). A more in-depth study found that it was well-tolerated at low doses (50–200 mg/kg) (Bloomfield et al., 1996; Przybelski et al., 1996), whereas some side effects, such as yellowing skin, hemoglobinuria, and jaundice, were observed at high doses (680–1,500 mg/kg). Taken together, DCLHb could be an alternative blood supply for patients with rare blood types or for emergency usage during the shortage of blood bank inventory (Reppucci et al., 1997; Schubert et al., 2002, 2003).

**Table 1:** Nanomedicines for the gas-carrier component.

**Components**	**Structure information**	**Applications**	**References**
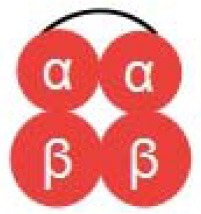	Crosslinking between two α subunits within a hemoglobin molecule	Hemorrhagic shock	Reppucci et al., 1997; Przybelski et al., 1999; Sloan et al., 1999; Schubert et al., 2002, 2003
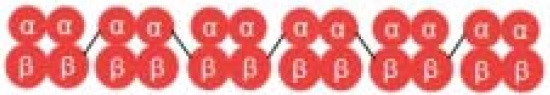	Crosslinking between adjacent hemoglobin α and β subunits	Emergency trauma and surgery Postoperative blood transfusion	Gould et al., 1998; Levy et al., 2002; Freilich et al., 2009
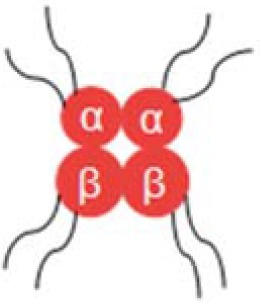	modified by PEG molecules	Blood transfusion Hemorrhagic shock	Nho et al., 1992; Bjorkholm et al., 2005; Olofsson et al., 2006; Vandegriff et al., 2008
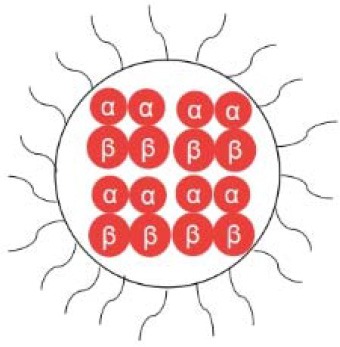	Nanostructure of outer PEG-modified lipid membrane	Blood transfusion Hemorrhagic shock	Sakai et al., 1997
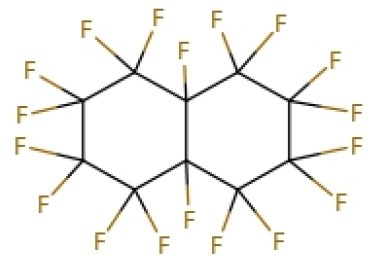	Perfluorocarbonated emulsions based on perfluorodecalin and egg yolk phospholipids	Acute shock of animals	Yokoyama et al., 1975
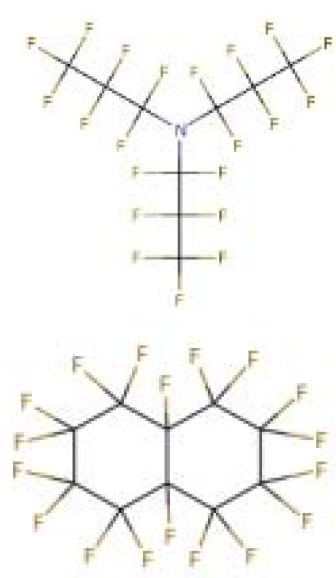	Fluosol-DA. Consists of perfluorodecalin, perfluorotripropylamine, hydroxyethyl starch and Krebs-Ringer bicarbonate	Increase patient oxygen delivery	Tremper et al., 1980, 1982; Suyama et al., 1981; Gould et al., 1986; Spence et al., 1994
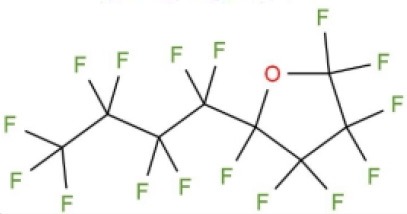	Perfluoro (2-n-butyltetrahydrofuran)	Provide oxygen for measuring nerve parts	Sanders and Schick, 1978
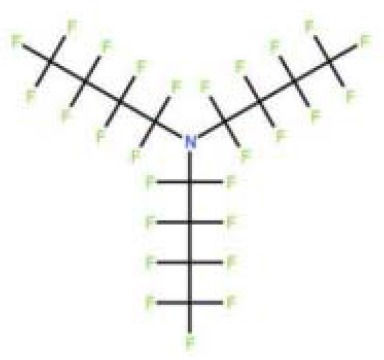	Perfluorotributylamine	Blood exchange	Motta et al., 1981
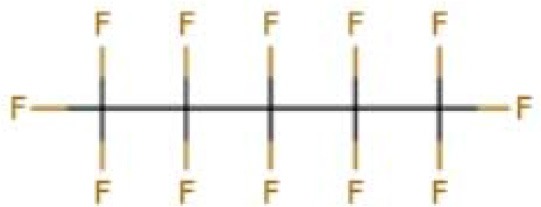	Dodecafluoropentane	Hemorrhagic shock Anemia Protection of the brain and heart	Woods et al., 2013; Borrelli et al., 2014; Moon-Massat et al., 2014; Strom et al., 2014; Culp et al., 2015
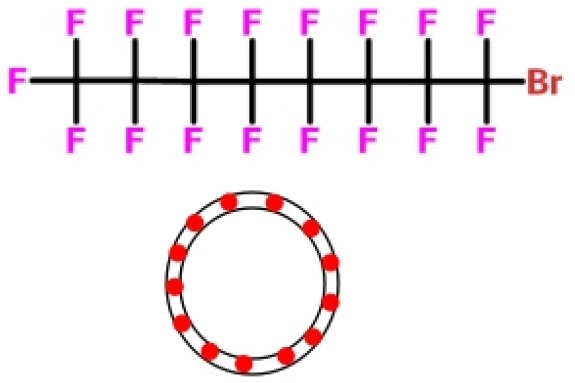	Perfluorooctane bromide	Auxiliary exchange gas	Pranikoff et al., 1996
	The modified hemoglobin is embedded in the liposome bilayer	Oxygen transport	Hasegawa et al., 1982; Tsuchida et al., 1984, 1988

Polymerized hemoglobin (PolyHeme, the structure is shown in Table 1) appeared to be a safer oxygen-carrying resuscitation fluid than DCLHb. It is a hemoglobin polymer complex formed by crosslinking adjacent α subunit and β subunit. In 1998, PolyHeme was first applied in trauma and emergency surgery, showing good tolerance and safety (Gould et al., 1998). Subsequent experiments showed that total hemoglobin level could be maintained by infusion of PolyHeme alone, thereby increasing the survival rate of patients with RBCs in critical situations (Gould et al., 2002). In addition to PolyHeme, researchers found that bovine aggregated hemoglobin (HBOC-201) was found to be able to keep up the hemoglobin level (Levy et al., 2002). In their work, a randomized, double-blind trial of HBOC-201 for patients undergoing heart surgery was conducted. After blood transfusion with HBOC-201, one third of patients survived without additional supply of RBCs. This indicates that HBOC-201 transfusion might be an alternative treatment for patients with moderate to severe anemia after cardiac surgery. Studies had shown that HBOC-201 is an effective oxygen carrier for most patients (Dong et al., 2006; Mackenzie et al., 2010); however, high vasoactivity-related adverse effects were found in the seniors with partial orthopedic surgery (Freilich et al., 2009).

Instead of cross-linking modification of hemoglobin, poly (ethylene glycol) (PEG) modified hemoglobin had attracted increasing attentions. For example, Nho et al. experimented with PEG-bovine hemoglobin in a low blood volume-bleeding shock model in dogs. The results showed that PEG-bHb has a longer half-life in blood vessels and no significant adverse effects compared to native hemoglobin (Nho et al., 1992). Björkholm et al. found that in a single-blind clinical trial of 12 volunteers they did not show any adverse symptoms at a dose of 50 mg/kg. When the dosage was increased to 100 mg/kg, the levels of amylase and lipase were slightly higher than normal (Bjorkholm et al., 2005). Hemospan is a PEG-modified human hemoglobin product developed by Sangart Inc and is also a widely used clinical preparation of RBCs substitutes. Eight PEG molecules were attached to the specific sites on α globulin and β globulin (Vandegriff et al., 2008), as depicted in Table 1. The purpose of this design is to prolong the retention time in blood vessel by increasing molecular weight, to increase the oxygen affinity, and to prevent it from early unloading (Vandegriff and Winslow, 2009). Olofsson et al. conducted a safety study in old patients undergoing selective hip arthroplasty and found that Hemospan is well-tolerated. No high frequency of adverse events from infusion to 24 h were observed along with a slight increase in liver enzymes and lipase (Olofsson et al., 2006). In a more recent study, Cooper et al. reported an PEGlated Hb engineered with tyrosine residues (Cooper et al., 2019), and it showed an increased vascular half time compared to wild type PEGlated Hb. The tyrosine residues were believed to enhance the reducibility of Hb and decrease adverse side effects, such as autooxidation.

Hemoglobin molecules were encapsulated in liposome particles (see Table 1), and the surface of the particles was attached with PEG-conjugated phosphatidylethanolamine (Sakai et al., 1997). Such HbV-PEG were found to be less prone to flocculation than unmodified hemoglobin vesicles. Animal experiments demonstrated that in comparison with the control group greater blood flow and faster gas exchange rates were observed for the group that had a 90% exchange transfusion with HbV-PEG/albumin (Sakai et al., 1997). Other Hb encapsulation protocols include polymersomes, hydrogels, or porous microparticles (Baudin-Creuza et al., 2008; Piras et al., 2008; Jia et al., 2016).

Devineau et al. reported a new strategy to form Hb-based oxygen carriers by directly adsorbing Hb onto the surface of silica NPs (Devineau et al., 2018). The authors claimed that the tetrameric structure of Hb was retained for carrying oxygen and the Hb-silica NPs complex has the potential to prevent the release of free Hb, which may lead to acute renal toxicity.

In summary, the development of nanomedicine for the application of Hb-based oxygen carriers should meet the four requirements (Benitez Cardenas et al., 2017): efficient oxygen delivery, low rates of NO scavenging, resistance to oxidative degradation, and high production yields. Chemical modification (such as cross-linking) and nano-carrier assistance are two main approaches to develop Hb-based nanomedicines for carrying oxygen. Prior to clinical trials, the latter requires more characterizations on structure and stability and more efforts to ensure the Hb-loading rates (Jansman and Hosta-Rigau, 2018). While site-specific mutations of Hb molecules had been engineered to enhance oxygen affinity, the intact Hb molecules that maintain the tetrameric structure were adopted and integrated into artificial nanostructures, either being attached onto nanocarrier's surface or encapsulated inside the nanocarrier. PEGlation provide a better bio-compatibility and enhance retention time. On one hand, molecular engineering would design protective pockets or formations to prevent oxidative molecules from penetrating the Hb, which leads to the degradation of Hb. On the other hand, adding antioxidation residues, such as tyrosine, into the mutated Hb molecule could lower the chance of autooxidation of Hb.

### Non-hemoglobin-Based Red Blood Cells Substitutes

#### Perfluorocarbon-Based Blood Substitutes

Perfluorocarbons (PFCs) emulsions are the first hemoglobin-free RBC substitutes discovered by scientists. PFCs were found to have oxygen and deoxygenation functions. Perfluoro compounds are saturated fluorocarbon molecules that have strong oxygen affinity. In early clinical practice, PFCs emulsions were administered intravenously directly or *via* a carrier and then left in the liver. However, some PFCs molecules hardly remain in the liver and are rapidly excreted through the breath and skin. Unlike hemoglobin-dependent RBC substitutes, PFCs solutions are white and are therefore referred as white blood.

In 1975, Yokoyama et al. first prepared a low-toxicity perfluorodecalin emulsion (the structure is shown in Table 1), which eliminated the disadvantage of the previous perfluoro compound that it accumulates in the body due to poor metabolism (Yokoyama et al., 1975). Subsequently, Naito et al. (Naito and Yokoyama, 1978) prepared a mixed emulsion, namely Fluosol-DA emulsion (the structure is shown in Table 1), containing perfluorodecalin, perfluorotripropylamine, hydroxyethyl starch, and Krebs-Ringer bicarbonate solution. It showed a significantly increase of the amount of oxygen delivered to the patient. Although it was prone to accumulate in the liver and spleen, it could be cleared by the lungs for a certain period of time (Tremper et al., 1980, 1982; Suyama et al., 1981; Spence et al., 1994). Fluosol-DA showed a high oxygen-carrying capacity, but its effectiveness appeared to be insufficient in certain critical situations, such as anemia patients with moderate and severe blood loss (Gould et al., 1986). Because of low durability and stability in the blood vessel as well as the complexity of configuration and use, continuing development of Fluosol-DA was hindered (Riess, 1992). Oxygent, i.e., perfluorooctyl bromide, is the second generation of PFCs emulsions and had been with a wide range of applications. Oxygent has more outstanding advantages than Fluosol-DA. First, it has a stronger oxygen carrying capacity. Second, it is more convenient to prepare and use without mixing different components. Third, it has a longer shelf life, i.e., it can be stored for more than 1 year at 5–8°C (Riess, 1992).

Keipert et al. investigated the feasibility of applying Oxygent as a blood substitute during surgery for patients under consciousness and anesthesia (Keipert, 1995). The results showed that in the state of continuous blood loss, the low level of Oxygent was able to ensure oxygen delivery, along with two side effects at high concentrations: body temperature rises (by 1–1.5°C in 4–6 h) and platelet count reducing. The results suggested that Oxygent could be used as a blood substitute for the patient of low-blood loss surgery. In addition to being an oxygen carrier, PFCs were found to selectively increase the radiation sensitivity of tumors, which could be beneficial for an adjuvant treatment of cancer (Teicher et al., 1992; Keipert, 1995). However, flu-like side effects were documented (Lane, 1995). In 1978, Sanders et al. synthesized a new PFCs compound, FC 75 (the structure is as shown in Table 1). Through potential measurement and program-induced hypoxia, FC-75 supplied oxygen to the nerves at the treatment site (Sanders and Schick, 1978). Motta et al. prepared an emulsion contains 20% perfluorotributylamine (see Table 1 for chemical structure) in 1981 for blood replacement in rabbits (Motta et al., 1981). Rabbit vital signs showed normal within 24 h of transfusion, and tissue analysis results indicated that it could act as a good oxygen carrier, despite of low toxicity.

Dodecafluoropentane (DDFPe, see Table 1 for chemical structure) is a special oxygen carrier with high oxygen affinity and transport capacity. It showed obvious protective effects on oxygen-dependent organs, such as brain and heart (Woods et al., 2013; Strom et al., 2014). Therefore, it has been developed for the treatment of brain damage and hemorrhagic shock (Moon-Massat et al., 2014). Intravascular fluorocarbon-stabilized microbubbles were reported to protect against fatal anemia in rats, since the oxygen carried in the microbubble was supplied for the brain functions for a certain period of time (Culp et al., 2015). As a carrier, DDFPe carries oxygen but also small molecule drugs at the same time. For example, thrombolytic drug tPA in a rabbit model of ischemic stroke or ischemic stroke (Nishioka et al., 1997; Borrelli et al., 2014). After targeted binding to blood clots suspended in the flow chamber, ultrasound was used to release oxygen or drugs. DDFPe mainly accumulates in the brain, but most of them can be removed within 2 h (Arthur et al., 2017). In a more recent study, Bonanno et al. investigated the efficacy of the DDFPe as an adjunct to prehospital resuscitation (Bonanno et al., 2018). The authors found that adding DDFPe into fresh frozen platelet does not improve survival or enhance tissue oxygenation.

The study of pulmonary mechanics in ventilator-assisted PFCs treatment in animals were conducted. In 1991, Fuhrman BP et al. used a conventional ventilator to add a normal residual volume of PFCs solution (30 ml/kg) to the piglet trachea. It was found that PFCs were able to directly participate in normal gas exchange in the lungs and did not cause significant adverse effects on piglets (Fuhrman et al., 1991). Experiments in lambs with respiratory distress syndrome have also demonstrated the feasibility of this approach. When using conventional ventilators, PFCs promoted the release of oxygen and carbon dioxide, thereby increasing blood oxygen levels in lambs (Leach et al., 1993).

In the treatment of respiratory failure in rabbits caused by saline lung lavage, the incidence and mortality rates were higher when treated with positive respiratory mechanical ventilation than that with a series of perfluoroether aeration treatments (Tutuncu et al., 1993). It had been found that adequate lung gas exchange could be maintained for hours at lower airway pressures. In 1996, Gauger et al. first conducted a PFC preliminary trial of children with acute respiratory distress syndrome (Gauger et al., 1996). The tested patients were six children extracorporeal life support (ECLS). After 2–9 days of ECLS, PFCs were added into the trachea and perform PFCs-filled lung gas ventilation (partial fluid ventilation). The cumulative amount of PFCs was 45.2 ± 6.1 mL/kg (range 30–72.5). The results showed that ECLS patients had a brief, normal respiratory exchange in the presence of PFCs. This indicates that PFCs can be safely used in the lungs of children with severe respiratory failure. Likewise, Pranikoff et al. investigated the biocompatibility of a perflubron (perfluorobromooctane) *via* a safety test in neonates with congenital diaphragmatics and severe respiratory failure (Pranikoff et al., 1996). PFCs was reported to enhance partial pressure of oxygen in animals with abnormal lungs (Hernan et al., 1994; Tutuncu et al., 1996). These studies have shown that PFCs can improve lung function and reduce the cost of intensive care for lung diseases.

PFC is a safe and mature oxygen carrier that can be directly chemically synthesized and has been widely used in medical treatment. As mentioned earlier, PFC molecules are generally emulsified to form particles (in micro level) and to be used as an oxygen carrier, and the emulsifier eventually decomposes in the body. The oxygen transport of PFC particles was well-known with a linear relationship between PaO_2_ and oxygen content, which contrasts with the sigmoidal oxygen dissociation of blood (Alam et al., 2014). Two main advantages of PFC-based oxygen transport to improve efficacy are: (a) PFC particles perfuse in the microcirculation of capillaries where no RBCs may flow; and (b) the oxygen carried by PFC particles is in dissolved state. Inspired by the PFC-based oxygen transport, PFC-base NPs were developed for multi-task nanomedicines, including bioimaging contrast agents and drug delivery carriers for diagnosis and treatment of diseases (Winter, 2014).

#### Heme-Based Blood Cell Substitute

Heme is a cyclic molecule composed of four pyrrole subunits surrounding a ferrous ion, which are most commonly recognized as one of the key components of hemoglobin. In the early studies, a ferrous porphyrin complex was embedded in a phospholipid bilayer of liposome to synthesize a liposome-heme complex (Tsuchida et al., 1988), as shown in Table 1. Tsuchida et al. converted the synthetic protohemoglobin into an amphiphilic heme molecule and encapsulated it in liposome. The liposome composite has a particle size of <0.1 micron. Compared to blood, the modified liposome heme has a faster oxygen reversible binding rate, a higher oxygen solubilization volume, and better stability. It also has smaller molecular weight than RBC. Artificial lung device test results indicate that the synthesized liposome heme can successfully deliver oxygen to muscle tissue (Tsuchida et al., 1988). The liposome heme molecule reversibly binds molecular oxygen in a neutral aqueous medium at 37°C. The oxygen adduct has an oxygen binding affinity (p 1/2) of about 50 mmHg and a half-life of half a day (Tsuchida et al., 1984). In addition, the iron (II) “picket fence porphyrin” complex with one hydrophobic imidazole is also incorporated into phosphatidylcholine molecules (the component of the phospholipid bilayer). Results have shown that The liposomal iron(II) porphyrin complex can be reversible with serum in the blood of rat at 25°C.

## Nanomedicines for the Immune-Responsive Component

In the blood, white blood cells (WBCs) are directly related to the immune regulation. WBCs can be deployed to the invasion site of the pathogenic microorganism, surrounded by phagocytosis, or the surface antigen being presented, and the signal is transmitted to the B cell or the T cell to exert the immune regulation function of the human body. BBDs caused by imbalances in WBCs often lead to the problems with immune response.

WBCs are a general term for a vast variety of immune cells, which generally include granulocytes, monocytes and lymphocytes. Among them, monocytes are precursors of macrophages. Macrophages have two common phenotypes, M1 (classically activated macrophage) and M2 (alternative lyactivated macrophage). M1 releases killing substances or pro-inflammatory factors from exogenous microorganisms or tumor cells, which promotes Th1 response. M1 has a distinct feature of high expression of IL-12 and low expression of IL-10. M2 repairs damaged tissues or releases anti-inflammatory factors, promoting Th2 response and expressing high IL-10 and low IL-12.

**Figure 1:**
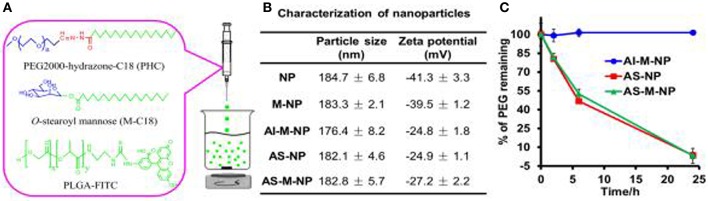
Preparation **(A)** and characterization **(B,C)** of PLGA-based nanoparticles (NPs). Reproduced with permission from (Zhu et al., 2013).

During the process of tumor growth, macrophages are mostly converted to the M2 phenotype that promotes tumor growth, and the nuclear factor-k-gene binding (NF-kB) signal is down-regulated (Xiang et al., 2018). The patient's immune system is unable to effectively recognize antigenic substances and initialize proinflammatory responses, thereby facilitating the proliferation and metastasis of tumor cells. Studies have shown that the differentiation of macrophages into the M2 phenotype may be caused by prostaglandin E2 (PGE 2) and interleukin 6 (IL-6) produced by tumor cells (Heusinkveld et al., 2011). However, tumor-induced M2 cells could be retransformed into activated M1 cells by stimulation (Heusinkveld et al., 2011). This provides a new idea for the treatment of tumors. M2 type macrophages (e.g., tumor-associated macrophages, TAMs) have been reported to overexpress the mannose receptor (Bhargava and Lee, 2012). Zhu et al. invented a nanoplatform targeting TAMs (Zhu et al., 2013). The preparation process and characterization results of the poly(lactic-co-glycolic acid) (PLGA)-based NPs are shown in Figure 1. The biodegradable PLGA NPs were modified with mannose (targeting TAM) and PEG, as shown in Table 2. The results indicate that the NPs can be taken up by normal macrophages and the TAMs *via* being targeted by mannose and its receptor after exfoliating PEG molecules under acidic microenvironment conditions (favorable for tumor). The platform has the potential to carry and deliver cancer drugs Doxorubicin (DOX) to treat triple-negative breast cancer (TNBC) (Niu et al., 2016). The results showed that a single intravenous injection of the NPs significantly reduced the M2 macrophage population in the tumor within 2 days, and the density of macrophages slowly recovered. It could be more effective if multiple injection of NPs or pre-treatment with zoledronic acid was carried out.

**Table 2:** Nanomedicines for the immune-responsive component.

**Structure**	**Configuration**	**Application**	**References**
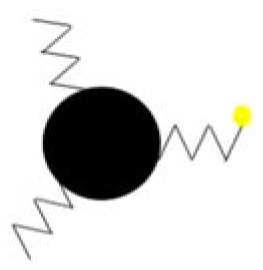	The composite NPs consisted of PIGA and the fluorescent group FITC, which was then modified with M-C_18_ and PEGylated under the action of PHC Black spheres: nanoparticles; fold line: the modification; and yellow sphere: the fluorescent group FITC	Targeting tumor-associated macrophages Targeted drug delivery (DOX) to tumor sites for the treatment of triple-negative breast cancer	Zhu et al., 2013; Niu et al., 2016
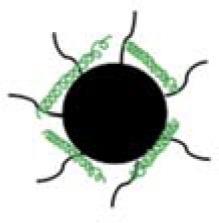	Polyamide-bonded NPs with a certain proportion of cholesterol molecules for siRNA delivery Black spheres represent nanoparticles. The black curve represents the cholesterol modification on the nanoparticles. The green helix is the siRNA carried by the nanoparticles	Targeting human breast cancer MCF-7 cell line and inhibiting tumor growth	Chen et al., 2013
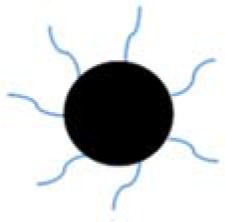	Carrying Dox and siRNA with synthetic PEAL polymer, and finally modifying with EGF Black spheres represent nanoparticles carrying siRNA and DOX. The blue curve represents the modification of the polyethylene glycol on the nanoparticles	Targeting mouse lung cancer cells, allowing cells to simultaneously take up siRNA and Dox, inhibiting the proliferation of cancer cells and the expression of Bcl-2 in tumor tissues	Zhang et al., 2016

**Figure 2:**
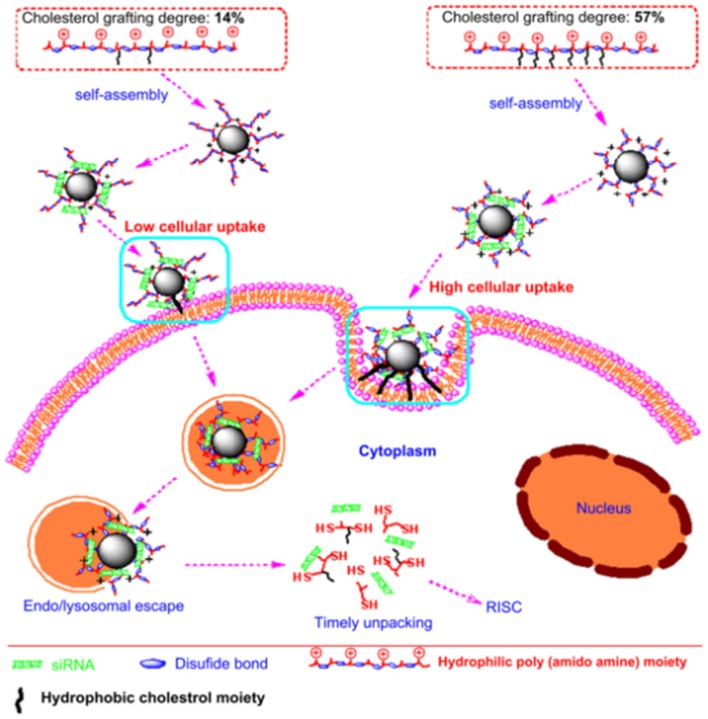
Nanostructured siRNA-coated and mediated gene silencing. The Poly(amidoamine)-cholesterol polymer forms a stable nanocomposite that was taken up by cells and had a strong *in vivo* inhibitory effect for tumor growth. Reproduced with permission from Chen et al. (2013).

Besides mannose, the targeting moiety could be cholesterol (Chen et al., 2013) or epidermal growth factor (EGF) (Zhang et al., 2016). Chen et al. used poly (amidoamine) to graft different percentages of cholesterol (rPAA-Ch) for siRNA delivery (Chen et al., 2013). The structure of NPs is shown in Figure 2 and Table 2. The results showed that the Poly(amidoamine)-cholesterol polymer forms a stable nanocomposite that was taken up by cells and had a strong *in vivo* inhibitory effect for tumor growth. Zhang et al. simultaneously delivered DOX and B-cell lymphoma-2-small interfering RNA(Bcl-2-siRNA) with an EGF-modified NPs (as shown in Table 2) to investigate their therapeutic effects on lung cancer (Zhang et al., 2016).

## Nanomedicines for Hemostasis-Responsive Component

Platelets are the smallest cells in the blood and have no nucleus, generated from megakaryocytes in the bone marrow. Molecular mechanisms of platelet-mediated hemostasis include primary and secondary hemostasis (Sen Gupta, 2017). During the primary hemostasis, platelets bind rapidly to specific proteins (e.g., von Willebrand Factor) and collagen at the bleeding site, followed by inter-platelet crosslinking *via* fibrinogen, which is recognized by the active GPIIb-IIIa on the surface of platelet molecules; on the phosphoserine-rich surface of active platelets, fibrin is formed and deposited in the process of coagulation cascade. Taken together, such coagulation process includes activation, adhesion and aggregation of platelets, as well as deposition and maturation of fibrin. Poor coagulation can cause thrombocytopenia, and hypercoagulability can lead to thrombosis. In a bleeding complication case, natural platelets or platelet-derived products are offered for transfusion. Such products suffer from short shelf-life, contamination, risks of infection/immunoreaction (unless prior serological testing was conducted). Artificial platelet-like biomaterials attract increasing attention to overcome such issues.

Platelet is of a micrometer size, loaded with thousands of biocomponents. Herein, scientists focus on the “platelet-inspired” nanomaterials that could function well as platelets during the hemostasis process and simplify the configuration of the nanomaterials. For instance, Jung et al. removed the content inside the platelet and obtained a natural aggregated platelet nanovesicles by using hypotonic ultrasound technology, which were then mixed with calcium chloride, thrombin, and membrane proteins to create a nano-aggregate (Jung et al., 2019) (as shown in Table 3). The nano-aggregate exert a hemostatic effect of platelets without causing an inflammatory reaction.

**Table 3:** Nanomedicines for hemostatic-responsive component.

**Main structure or composition**	**Configuration**	**Application**	**References**
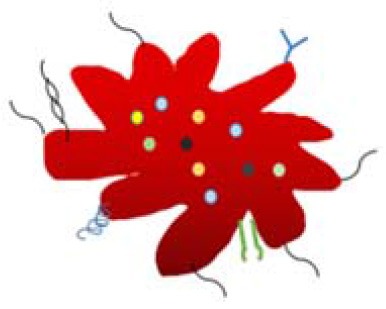	Platelet-simulated NPs containing various ligands, small molecules, proteins and liposomes Radiation star when activated Various colored particles indicate that the simulated platelets contain multiple proteins and liposome small molecules (e.g., Cell mitogens, Hemostatic components, Adhesion molecules and antigens). A variety of ligands and modifications are modified on the surface of the nanoparticles (e.g., TP receptor and P-selectin)	Target cancer cells with certain adhesion	Modery-Pawlowski et al., 2013; Jung et al., 2019
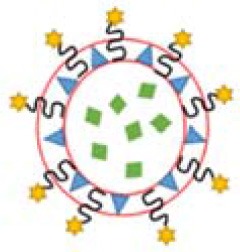	TRAL-Dox-PM-NV Dox-coated and TRAL modified on natural platelet vesicle membrane surface-modified platelet NPs Hollow round with blue double layer as platelet membrane. Green diamonds for DOX in hollow round interior. Yellow stars are modified by the platelet membrane to indicate TRAIL	Target tumor cells and induce apoptosis	Hu et al., 2015
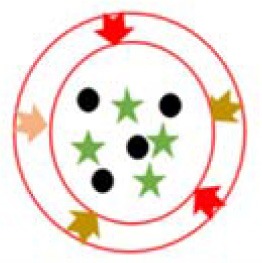	RGD peptide modified encapsulated melanin NPs and Dox nanoscale platelet vesicles Arrows represent RGD peptides modified on the surface of platelet nanoparticles. Black spheres represent melanin molecules Green stars represent DOX	Targeting tumor cells, supplemented by subsequent treatment, can alter multidrug resistance of tumor cells	Jing et al., 2018
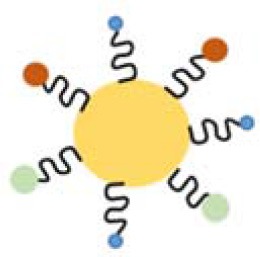	Protein or small molecule is modified on the surface of liposome NPs Yellow spheres as liposome nanoparticles. Red and green spheres represent different protein molecules modified on the nanoparticles, such as surface conjugated linear Arg-Gly-Asp (RGD) peptide moieties, fibrinogen gamma-chain. The blue sphere is represented as a small molecule modified on the nanoparticle. Such as 3′-O-Sulfated Le (a) [SO3Le (a)], integrin α (IIb) β (3), P-selectin	Delivery of thrombolytic drugs to prevent platelet aggregation	Gupta et al., 2005; Yan et al., 2005; Modery et al., 2011; Absar et al., 2013; Pan et al., 2017; Huang et al., 2019
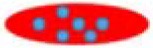	Inject small molecules directly into platelets by electroporation or other methods Natural platelet molecules are represented by a red ellipsoid. The blue sphere is represented by a small molecule such as DOX that is coated in platelets	Delivery of drugs Reduce platelet aggregation	Ward et al., 1995; Xu et al., 2017

Recent developing trend would be from enhancing platelet adhesion and hemostatic plug formation to harness more platelet's functions, such as clot contraction, and to build a minimal system, i.e., artificial platelet (Majumder and Liu, 2017), which will be able to perform some essential functions of natural platelet, including vesicle encapsulation, attachment, fusion, and protein production. Platelet-like particles (PLPs) that are coupled to fibrin-binding antibodies had shown the capability to mimic natural platelet functions to bind the wound site, stabilize clot structure, and enhance clot formation (Brown et al., 2014). Further improvements enabled a core-shell PLP to facilitate temporal control over clot retraction (Sproul et al., 2018) and mimic the antimicrobial action of platelets by integrating with gold nanoparticles (Sproul et al., 2019), which may improve healing outcomes after hemostasis. A synthetic liposomal platelet surrogate, Syntho Plate, has shown bifunctional features, i.e., adhesion and aggregation, thanks to the integration of three peptides: von Willebrand Factor-binding peptide, collagen-binding peptide, and GPIIb-IIIa-binding fibrinogen-mimetic peptide (Shukla et al., 2017). The Syntho Plate was applied to animal testing after femoral artery injury, and the result showed that blood loss was reduced, blood pressure was stabilized, and survival rate was improved (Hickman et al., 2018).

**Figure 3:**
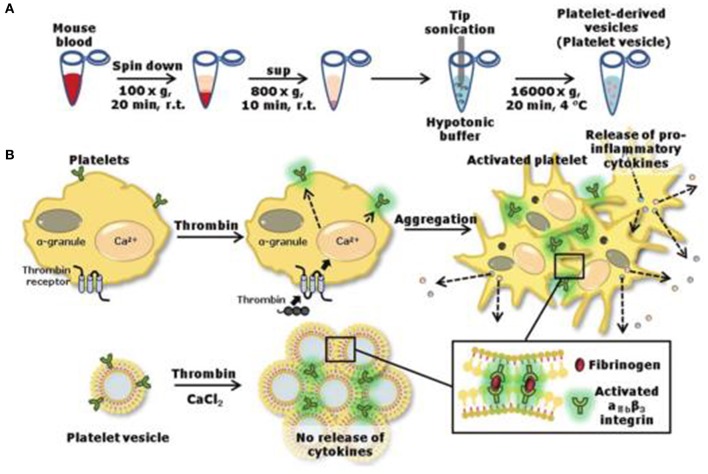
Preparation **(A)** of nanovesicles and release **(B)** of pro-inflammatory factors. Reproduced with permission from Jung et al. (2019).

On the other hand, thrombosis, an abnormal aggregation of platelets, is highly likely to occur after surgery, such as coronary arteries. Ward et al. perforated platelets with high-pressure discharge, added ilocprost (drug) into the extra-platelet membrane, and then closed the membrane pores in a 37-degree warm bath for 40 min. Experiments in rabbits of atherosclerosis have shown that modified platelets can reduce platelet deposition by 64% in comparison with native platelets (Ward et al., 1995). Recently, platelet-targeting peptides- or small molecules-modified micelles/liposomes, shown in Table 3, were reported to deliver thrombolytic drugs to prevent platelet aggregation (Gupta et al., 2005; Yan et al., 2005; Modery et al., 2011). Pan et al. developed a Amnexin V-conjugated micelle that was synthesized with biodegradable polymer and loaded with lumbrokinase for phosphatidylserine thrombolysis (Pan et al., 2017). *In vitro* experiments have shown that such micelles are much more efficient in thrombolysis than the control group. *In vivo* experiments show that the micelles have good capability of targeting and thrombolysis. A PEGylated liposome, modified with a peptide of fibrinogen gamma chain (CQQHHLGGAKQAGDV) and loaded with thrombus-specific tissue plasminogen activator (tPA), was developed by Absar et al. (2013). *In vitro* and *in vivo* rat thrombolysis experiments demonstrated that the nano-liposomal vector targets activated platelets and rapidly releases tPA. Compared with natural tPA, such tPA-liposome complex increased the thrombus solubility by 35% and reduced the consumption of circulating protein fibrinogen by 4.3 times, which greatly improves the thrombolysis efficiency. Huang et al. synthesized a tPA-loaded PEGylated liposome, approximately 164.6 ± 5.3 nm in diameter (Huang et al., 2019). As shown in Figure 3, the surface was coated with cyclic arginine-glycine-aspartate (cRGD). The presence of activated platelets allows synthetic nanoliposomes to induce efficient fibrin clot lysis in the fibrin-agar plate model, and the concentration of activated platelets determines the extent of tPA release. The liposome membrane protected well the activity of tPA (retaining 97.4 ± 1.7% fibrinolytic activity) to achieve efficient thrombolysis.

On February 6, 2019, FDA approved Cablivi (caplacizumab-yhdp) injection (FDA, 2019a) in combination with plasma exchange and immunosuppressive therapy for the treatment of adult patients with acquired thrombotic thrombocytopenic purpura (aTTP), a rare and life-threatening BDD. Patients with aTTP develop extensive blood clots in the small blood vessels throughout the body. Caplacizumab is a humanized, bivalent, variabledomain-only immunoglobulin fragment, named “Nanobody” by Ablynx. It targets the A1 domain of von Willebrand factor, preventing interaction with the platelet glycoprotein Ib-IX-V receptor and the ensuing microvascular thrombosis (Callewaert et al., 2012; Scully et al., 2019).

## Platelet-Inspired Nanomedicines

The development of new nanomedicines, inspired by platelet, is beyond the two above-mentioned chapters. Some studies have shown that there is an interaction between platelets and tumor cells. The formation of platelet-tumor cell aggregates and their sequestration in various end-organs may result in thrombocytopenia (Karpatkin and Pearlstein, 1981; Mehta, 1984). In addition, platelet-derived factors secreted by platelets stimulate the proliferation of tumor cells, while secreting certain substances to increase vascular permeability is beneficial to the metastasis of tumor cells (Gasic et al., 1973; Karpatkin and Pearlstein, 1981; Karpatkin et al., 1988; Schumacher et al., 2013). Therefore, the unique interaction between platelet and tumor cells could be utilized by scientists for developing anti-cancer nanomedicines.

**Figure 4:**
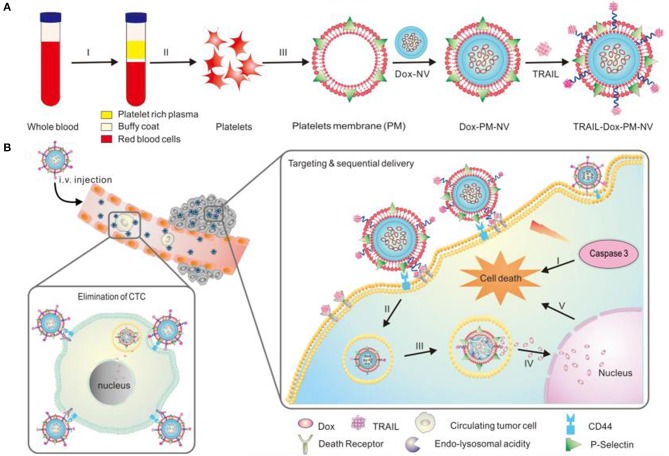
Schematic design **(A)** of drug-loaded PM-NV for targeting and sequential drug delivery **(B)**. The functionalized nanocarrier targeted tumor cells and induced apoptosis. Adapted with permission from Hu et al. (2015).

Modery-Pawlowski et al. designed a platelet mimetic (as shown in Table 3), loaded with ligands and other components, in order to investigate the interaction between such complex and metastatic cancer cells (Modery-Pawlowski et al., 2013). The results indicated that platelet mimics bind to cancer cells and exhibited greater cell targeting ability and adhesion for metastatic human breast cancer cell line MDA-MB-231 than cell line MCF-7. Hu et al. encapsulated the tumor-killing small molecule drug, Dox-NV, in a tumor necrosis factor-related apoptosis inducing ligand (TRAIL)-coated nanocarrier (TRAIL-Dox-PM-NV, as shown in Table 3) (Hu et al., 2015) that is formed with a platelet nanofilm, which was obtained by removing the internal contents. The results showed that the functionalized nanocarrier targeted tumor cells and induced apoptosis, as shown in Figure 4.

Jing et al. designed a nanoparticle, RGD-NPVs@MNPs/DOX (as shown in Table 3), for multidrug resistance (MDR) cancer and performed *in vitro* experiments using different cell lines (Jing et al., 2018). The mixture of melanin NPs (MNPs) and RGD-modified platelet membrane was extruded by ultrasound to form a MNPs-loaded RGD-coated nano-platelet vesicle, followed by loading with DOX. The results showed that such vesicle can target and kill tumor cells.

Synthetic NPs may cause cytotoxicity to endothelial cells (Ilinskaya and Dobrovolskaia, 2013). Natural platelets therefore became an ideal drug delivery vehicle. Xu et al. encapsulated DOX in platelets through an open tubule system to make a DOX-platelet complex (as shown in Table 3) and conducted a series of experiments on Raji cells in the tumor-bearing mouse model (Xu et al., 2017). Compared to free DOX, DOX-platelets were able to target tumor cells and release more DOX at an acidic pH; the DOX-platelet stayed longer in the blood (half-life t1/2 = 29.12 ± 1.13 h). Tissue distribution results showed that DOX released by DOX-platelet mostly exists in tumor cells, along with low residuals in the organs, such as heart, liver, spleen, kidney, and lungs. This indicates a strong anti-tumor effect.

## Conclusions and Outlook

Nanomedicines is a hybrid term of nanotechnology and medicine, converting traditional small molecule medicine into a nano-sized carrier loaded with drug molecules or other therapeutic (bio)moieties and delivery system for disease treatment. As for BDDs treatment, a potential application of nanomedicines is to offer artificial blood components, which may possess some advantages over blood: long shelf-life, good stability, and large-scale availability. On one hand, engineered or modified hemoglobin molecules, as well as hemoglobin-loaded liposome, were reported to serve as RBC substitute to carry oxygen. On the other hand, PFCs- or heme-based non-hemoglobin substitutes show different oxygen disassociation factors. The findings in the development of PFCs-base substitutes provide a mechanism guideline for future design of multifunctional PFCs nanomedicines (carrying oxygen, drug delivery, and bioimaging), as the PFCs nanoparticles had been approved by FDA for contrast agent in clinical applications. It has been realized that nanomedicines cannot replace the exact same role as the RBCs because the complicated components of the latter and the size difference. Nanomedicines may mimic the main features of the RBCs by integrating the functioning components into the nanocarrier. Likewise, artificial nanomedicines were designed to mimic the specific affinity (or binding) of WBCs or platelet in order to act as an immune- or hemostasis-responsive component. Inspired by the biocompatible platelet membrane, platelet-like functional nanomedicines were developed and showed better drug delivery efficacy.

While scientists are dedicated to make “blood” *ex vitro* through stem cell research, it is also important to design nanomedicines for BDDs treatment. Researchers are trying to close the gap between artificial nanomedicine and natural blood components. Not only the potential toxicity of nanomedicines (e.g., activating immunoreaction), but also the complications of scale-down (from micro-size cell to nano-size system) and simplification of functioning components that may result in the lack of some comprehensive functions, shall be under consideration.

Some new drugs for BDDs treatment had been approved by FDA, including Elzonris (tagraxofusp-erzs) for BPDCN, Cablivi (caplacizumab-yhdp) for aTTP, and Jakafi (ruxolitinib) and Inrebic (fedratinib) for myelofibrosis (FDA, 2019b). These had set good examples for researchers, academic and industrial, to get a better understanding how to move forward the development and (pre)clinical trial of nanomedicine for BDDs treatment. It was 1995 that FDA approved the first nanomedicine (NPs drug), namely Doxil, yet no nanomedicines were approved for BDD treatment. A straightforward path will be to integrate the approved drugs for BDDs treatment into a nanocarrier, forming a promising candidate of nanomedicine.

A critical step to move forward is from preclinical testing to clinical trial. Preclinical testing focuses mainly the characterization of nanomedicine. Prior to clinical trial, another challenging step for nanomedicine development for BDD treatment, as well as for other tumor/cancer treatment, is the scale-up manufacturing. It was estimated that an ~8% passing rate for nanomedicines, but only one fourth for small molecules drugs (Torrice, 2016). When used “blood disease” and “liposome” or “nanoparticles” to search on Clinicaltrials.gov, we obtained more than 150 open or active clinical trials. This is an exciting news for researchers working in this field.

A debating issue of nanomedicine development is the drug delivery efficiency of nanomedicines provoked by Chan's group (Wilhelm et al., 2016) in 2016, which documented that the number of nanomedicines reaching the tumor site was <1%. Some researchers or drug-makers argued that the retention time and maximum concentration at the tumor site are more important than the efficiency, and they, especially from a more practical perspective, were more intrigued to find ways to minimize the impact of nanomedicines on non-target organ (Torrice, 2016), such as liver. Information gathered from this debate will also be an inevitable step for the nanomedicine development for BDDs treatment.

## Author Contributions

M-YW and QM oversee the content and writing of the manuscript and reviewed the finalized the manuscript. NZ wrote the draft.

### Conflict of Interest

The authors declare that the research was conducted in the absence of any commercial or financial relationships that could be construed as a potential conflict of interest.
